# Three-dimensional collimated self-accelerating beam through acoustic metascreen

**DOI:** 10.1038/srep17612

**Published:** 2015-12-01

**Authors:** Yong Li, M. Badreddine Assouar

**Affiliations:** 1 CNRS, Institut Jean Lamour, Vandoeuvre-lès-Nancy F-54500, France; 2 Université de Lorraine, Institut Jean Lamour, Boulevard des Aiguillettes, BP: 70239, Vandoeuvre-lès-Nancy 54506, France

## Abstract

We report the generation of three-dimensional acoustic collimated self-accelerating beam in non-paraxial region with sourceless metascreen. Acoustic metascreen with deep subwavelength spatial resolution, composed of hybrid structures combining four Helmholtz resonators and a straight pipe, transmitting sound efficiently and shifting fully the local phase is evidenced. With an extra phase profile provided by the metascreen, the transmitted sound can be tuned to propagate along arbitrary caustic curvatures to form a focused spot. Due to the caustic nature, the formed beam possesses the capacities of bypassing obstacles and holding the self-healing feature, paving then a new way for wave manipulations and indicating various potential applications, especially in the fields of ultrasonic imaging, diagnosis and treatment.

There are amount of requirements to control wave fields with desired patterns, such as non-diffracting^1^, twisted^2^ wave front. One of the most intriguing phenomena which attracted considerable research interest recently is the notably self-accelerating beam since the concept of Airy beam was introduced for optical wave^3^,^4^,^5^,^6^,^7^. These realization of self-accelerating beams in paraxial and non-paraxial domains propagating along designed trajectories indicates amount of potential applications, such as guiding micro-particles^8^, producing curved plasma channels^9^, and so on. In principle, these self-accelerating beams are formed based on the special solutions of wave equations or caustic theory^10^.

As another classic wave, acoustic wave obeys the Helmholtz wave equation, indicating the possibility that it can be designed to propagate along desired trajectories. Recently, acoustic self-accelerating beam were demonstrated both numerically and experimentally with *active* phased arrays^11^,^12^. However, the sources in the *active* way require to be operated individually with electric techniques, resulting in the high cost and complexity. To avoid these significant limitations, considerable efforts have been dedicated to exploring the *passive* control of sound by means of the metasurface^13^,^14^,^15^,^16^,^17^,^18^,^19^,^20^ or metascreen^21^, which can be regarded as ultra-thin metamaterials^22^,^23^,^24^,^25^. To form self-accelerating beam in non-paraxial domain with excellent performance, the *passive* structures should possess the capacities of transmitting sound energy effectively, shifting the phase of incident wave covering 2*π* range, and holding a subwavelength feature to avoid the spatial aliasing effect^26^. These conditions are rarely realized simultaneously by the previous models, resulting in the fact that the non-paraxial self-accelerating beams and their physical features and potential applications were rarely explored. Furthermore, all the previous models are designed in two-dimensional space, which inevitably hinder the real applications. Actually, three-dimensional acoustic self-accelerating beams, if realized, could open a new degree of freedom for acoustic wave manipulations and have deep implications in acoustical applications where special control of sound is needed. For instance, the unique self-healing behavior of the beam could provide a promising solution to the narrow “acoustic window” resulting from the obstruction of the rib cage in ultrasonic ablation of liver tumors.

Here we present the generation of a three-dimensional acoustic collimated self-accelerating beam with sourceless metascreen. By imposing a fine local phase shift profile on the metascreen, the sound energy could be delivered along a designed curved trajectory and then focused at a spot even with existing blocking obstacles in front of the spot and along the trajectory.

## Results

### Illustration

**Figure 1:**
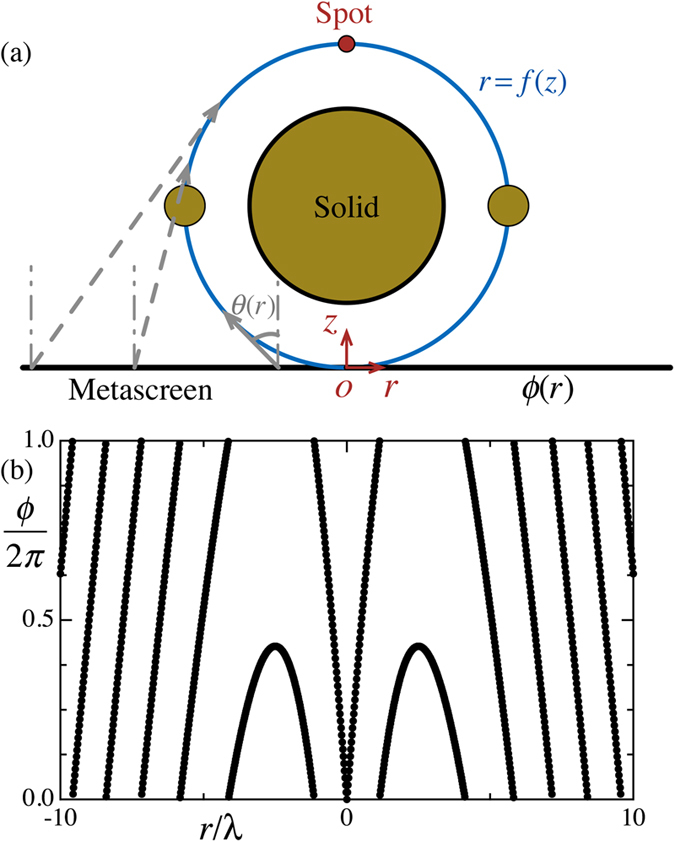
Illustration of a three-dimensional collimated self-accelerating beam. (**a**) The metascreen (black line) in a cylindrical coordinate can provide a local phase shift profile *ϕ*(*r*) on an incident wave to transmit sound propagating along a curved trajectory *r* = *f*(*z*) (blue line) and focusing at a spot (red dot). For the demonstration the capacities of the metascreen, a spherical and a ring-like solid obstacle (three yellow circles in the *r* − *z* plane) are placed in front of the metascreen and along the trajectory to block the formation of the focused beam. (**b**) The required phase shift profile provided by the metascreen to form a self-accelerating beam propagating along a circular trajectory with diameter of 2.5*λ*. The phase curve covering 2*π* range varied rapidly along the *r* direction.

The desired three-dimensional acoustic collimated self-accelerating beam in non-paraxial domain is illustrated in Fig. 1(a). The metascreen possess the abilities of providing a local phase shift *ϕ*(*r*) on the incident sound field, consequently shaping the transmitted sound propagating along a desired trajectory *r* = *f*(*z*), and finally forming a focusing spot at a the intersectional region of the trajectory. The relationship between the phase shift profile and the desired trajectory could be retrieved from tracing each individual caustic ray and expressed as^11^,^12^,^27^


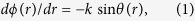


where *k* is the sound wave number in the medium and *θ*(*z*) is the angle of the path [cf. Fig. 1(a)]. Using this relation, the desired phase profile *ϕ*(*r*) can be calculated by finding the inverse tangent of the path slope


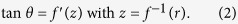


As an example of the self-accelerating beam beyond the paraxial approximation, we employ a circle trajectory


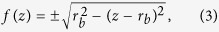


with center at (*r*, *z*) = (0, *r*_*b*_). The desired phase shift profile for forming such a bending beam from a normally plane wave is


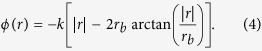


Figure 1(b) shows the desired phase profile for the forming of a circle beam with *r*_*b*_ = 2.5*λ*. This phase profile illustrates the requirement of the phase shift profile provided by the metascreen for the desired beam with good performance. The requirement is the ability of providing a phase shift that can span a full 2*π* range in a controllable manner and rapidly varies along the metascreen in *r* direction^11^,^21^. The variation is in a subwavelength scale so that the metascreen needs to hold a fine spatial resolution when using discrete structures along *r* directions to avoid spatial aliasing effect.

To illustrate the performance of the self-accelerating beam, we will place a spherical and a ring-like obstacle in front of the metascreen and along the propagating trajectory to obstruct the formation of the desired focused wave field. The big circle and the two small ones [cf. Fig. 1(a)] in the *r* − *z* plane refer the spherical and the ring-like obstacle, respectively.

### Design

**Figure 2:**
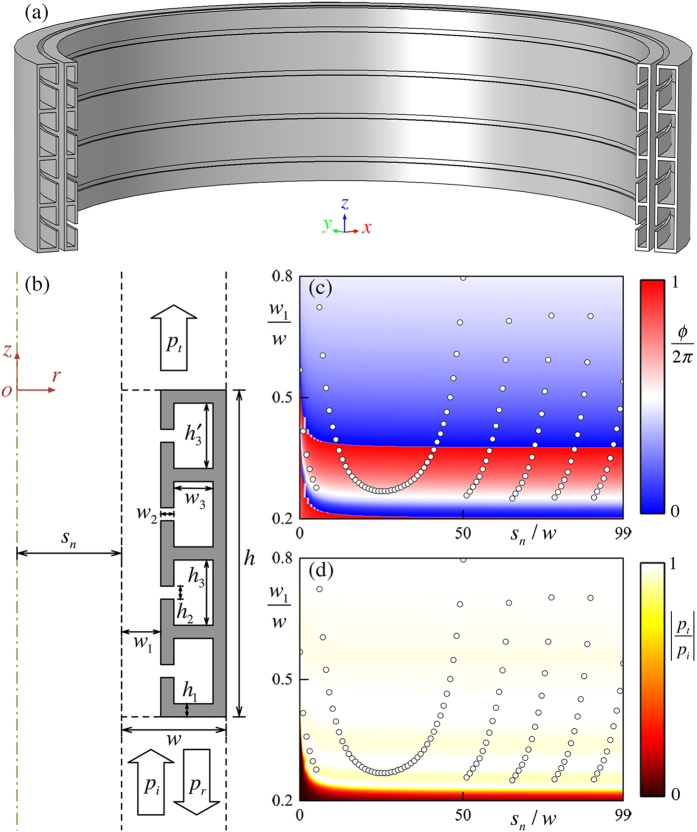
The metascreen is constructed by a series of individual elements. (**a**) Three-dimensional configuration of two adjacent axisymmetric elements. Incident wave propagating along +*z* direction penetrates metascreen through the slit (straight pipe) between the adjacent elements. (**b**) Schematic digram of an individual element in *r* − *z* plane (width *w* = *λ*/10 and height *h* = *λ*/2) consisting of four Helmholtz resonators (HRs) and a straight pipe (width *w*_1_). The width and height of the throat (cavity) of the HRs are *w*_2_ (*w*_3_) and *h*_2_ (*h*_3_). The HRs are formed by solid materials with identical height *h*_1_ = *w*_2_. The distance from the individual element to the axis is *s*_*n*_, which is the integral multiple of *w*. (**c**) The phase shift map and (**d**) the transmission coefficient map of the presented metascreen as functions of *s*_*n*_/*w* and *w*_1_/*w*. The geometric parameters in the simulations are fixed as (*h*_1_, *h*_2_, *h*_3_, 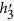) = (0.01, 0.03, 0.235, 0.24)*h*. The hollow circle point indicates the *w*_1_ and *s*_*n*_ of the elements achieving the desired phase profile from Eq. 4 with *r*_*b*_ = 2.5*λ*.

To realize the desired phase profile shown in Fig. 1(b), we use a three-dimensional subwavelength hybrid elements to construct the metascreen. Figure 2(a) illustrates two adjacent elements in three-dimensional space to demonstrate the configuration of the metascreen. Figure 2(b) shows an individual element in *r* − *z* plane consisting of four Helmholtz resonator (HRs) and a straight pipe. Here the series connection of the HRs acts as acoustic resistance to shift the phase of the incident wave^28^. The cavity series has a tunable width *w*_3_ to span the phase shift over a full 2*π* range. The functionality of the straight pipe with fixed length of *λ*/2 supporting Fabry-Pérot resonance^29^,^30^,^31^ is to provide coupling resonances keeping relatively impedance matching^21^. Considering the fact that the transmission coefficient is determined by the coupling resonances between the Fabry-Pérot and Helmholtz resonance, the number of the HRs is selected to be four in order to provide enough coupled resonances so that transmission coefficient can keep high value while covering 2*π* range^21^.

Distinct to the models in two-dimensional cartesian coordinates where the transmission coefficient, |*p*_*t*_/*p*_*i*_|, and phase shift, *ϕ*/(2*π*), is independent of the position of the individual element, these variables in cylindrical coordinates are related to the distance from the element to the axis, *s*_*n*_. This difference stems from the different volumes of the elements locating at different *s*_*n*_ even with identical geometrical parameters (such as *w*_1_, *w*_3_). Figure 2(b,c) illustrate the simulated phase shift and transmission coefficient map as functions of the straight pipe width ratio, *w*_1_/*w* and the distance ratio, *s*_*n*_/*w*. Due to the symmetric geometry, only the positive part where *r* ≥ 0 is illustrated. The phase shift could cover 2*π* range for each elements (viz., 0 ≤ *s*_*n*_/*w* ≤ 99) when tuning 0.2 ≤ *w*_1_/*w* ≤ 0.8, even the one for individual elements changed slightly with different *s*_*n*_. Furthermore, the transmission coefficient is considerably high where the phase shifts covering 2*π*.

According to the Fig. 2(b), it is readily to obtain a required phase shift profile for a corresponding beam. For example, we choose the radius of the circular trajectory as *r*_*b*_ = 2.5*λ*. In order to obtain a good shaping of the desired beam, the number of the individual elements composed of the whole metascreen is fixed to be 100. Then the *w*_1_/*w* needed for the desired phase shift profile [cf. Fig. 1(b)] is illustrated as hollow black point [cf. Fig. 2(b,c)]. It can be found that the metascreen can transmit sound with high efficiency greater than 91% and shift the incident phase covering 2*π*. The spatial resolution of the metascreen, viz., *w*, is as small as *λ*/10, which is fine enough to avoid the spatial aliasing effect.

### Collimated self-bending beam

**Figure 3:**
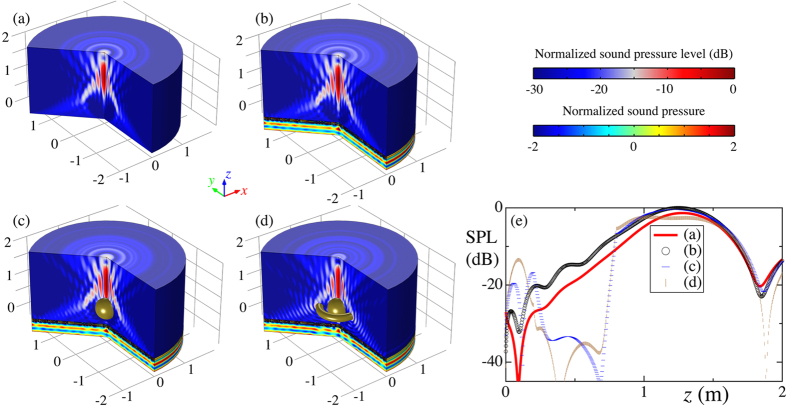
Collimated self-bending beam from a normally incident plan wave. (**a**) The formed collimated self-bending beam with the desired phase shift profile with *λ* = 0.2 m. (**b**) The sound field of the metascreen constructed by 100 elements, where the field at *z* > 0 is sound pressure level (SPL, normalized by the maximum value) and at *x* < 0 is a snapshot of normalized sound pressure. The metascreen yields a phase profile *ϕ*(*r*) on the normally incident plane waves propagating along +*x* direction. (**c**) Sound field same to (**b**) while with a spherical obstacle centered (*r*, *z*) = (0, *r*_*b*_/2) (yellow region, diameter *d*_*s*_ = 3*λ*) in front of the metascreen. (**d**) Sound field same to (**c**) while with an additional ring-like obstacle centered (*r*, *z*) = (*r*_*b*_/2, *r*_*b*_/2) (yellow region, cross-sectional diameter *d*_*r*_ = *λ*) located along the trajectory. (**e**) Comparison of the SPL along the axis in (**a**–**d**). The SPL is normalized to the maximum value along the *z* axis. A focused spot could be observed near *z* = 6.3*λ*. The large deviation of the SPL around *z* = 0.5 m convincingly stems from the existing spherical obstacle.

The realization of our screen allows effective control of sound propagation along desired trajectory. The desired collimated self-bending beam is shown in Fig. 3(a). A boundary with a unity amplitude and a continuous phase profile is employed to form the self-bending beam. We construct the metascreen with 100 elements along *r* direction with desired geometrical parameters, *w*_1_/*w* and *s*_*n*_/*w*, shown in Fig. 2(b,c). The transmitted wave fields through the metascreen is shown in Fig. 3(b) with a normally plane incident wave propagating along +*z* direction. The screen yields a discrete desired phase shift profile on the incident wave with spatial resolution *w* = *λ*/10. The self-bending beam is well established [cf. Fig. 3(b)] and in a good shape of the desired propagating trajectory and then focused at the spot. Excellent agreement could be obtained by comparing the wave fields in Fig. 3(a,b). The excellent performance of the proposed metascreen owes to the fine spatial resolution, the high transmission and the fully controlled phase shift.

The self-bending beam possesses the capacity to bypass solid obstacle due to the curved trajectory. From Fig. 3(c), one can observe that transmitted field pattern nearly keeps identical even with the existing scattering from the solid spherical obstacle (diameter 3*λ*) located in the region surrounded by the main lob. Additionally, the metascreen holds its own self-healing feature. A ring-like obstacle (diameter *λ*) located along the trajectory that blocks the main lob of the beam is added. The beam restores to its shape [cf. Fig. 3(d)] after passing the obstacles and forms the desired focused spot. In order to qualify the observed features, a comparison of the normalized sound pressure level (SPL) along the *z* direction [cf. Fig. 3(e)] shows that, even when both obstacles simultaneously occupy the space, the beam endows extremely robust against perturbations, owing to its caustic nature.

Our metascreen not only can transmit normally incident plane waves but also any sound fields to form desired beams in homogeneous medium. Due to the fine spatial resolution of the metascreen, the width of the inlet and outlet of the individual element, *w*_1_, is in deep subwavelength scale so that the pressure along the *r* direction in these regions could be regarded as a uniform value. The designed metascreen should provide another phase shift profile to compensate the phase difference along the boundary in the incident side. As an example, a point source located at (*r*_*s*_, *z*_*s*_) = (0, −10*λ* − *h*) is employed to radiate a spherical wave. To form the same non-paraxial self-accelerating beam, the local phase shift provided by the metascreen can be expressed as


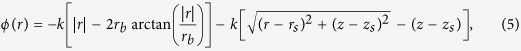


where the second part with *z*=-*h* compensates the arrival phase difference of the point source along the boundary of the metascreen at the incident side. While the first part is same to Eq. 4.

**Figure 4:**
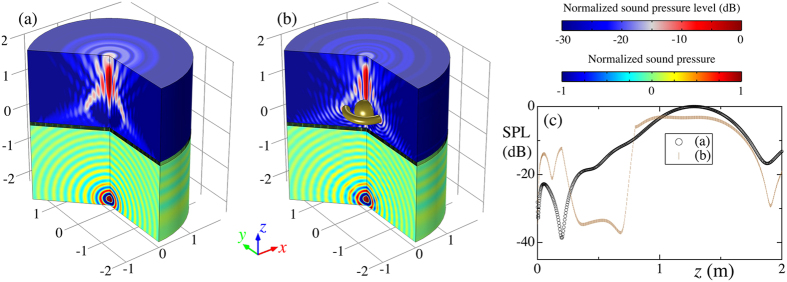
Collimated self-bending beam from a point source. (**a**) The radiated sound field (normalized pressure field) from a point source located at (*r*_*s*_, *z*_*s*_) = (0, −10*λ* − *h*) and the transmitting one (normalized sound pressure level) through the metascreen with *λ* = 0.2 m. (**b**) Sound fields same to (**a**) while with a spherical obstacle in front of the metascreen and a ring-like obstacle along the trajectory. (**c**) Comparison of the SPL along the *z* axis in (**a**,**b**). The large deviation of the SPL around *z* = 0.5 m convincingly stems from the existing spherical obstacle.

The realized collimated self-bending beam from the point source [cf. Fig. 4(a)] propagating along the designed trajectory closely resembles the desired beam illustrated in Fig. 3(a), providing a solid support for the great capacity of our presented screen. It is also not surprising to observe that the non-paraxial accelerating beam can convincingly bypass solid obstacle due to the curved trajectory and hold its own self-healing feature [cf. Fig. 4(b)]. A comparison of the SPL along the *z* direction for these cases [cf. Fig. 4(c)] indicates that, even if both obstacles block the formation of the desired wave field, the self-bending beam could be reconstructed to propagate along the desired trajectory and focused behind the solid obstacles.

## Discussion

In conclusion, we have proposed a three-dimensional acoustic metascreen constructed by combining a series connection of four Helmholtz resonators with a straight pipe supporting Fabry-Pérot resonance. The elements of the metascreen can effectively transmit sound energy, steer the phase shift covering a full 2*π* range and hold a fine spatial resolution in *r* direction as small as *λ*/10 to avoid the spatial aliasing effect. With these great capacities, acoustic metascreen composed of 100 individual elements along the *r* direction was implemented to generate collimated non-paraxial self-bending beams, whose self-healing and bypassing behaviors were further demonstrated.

The realization of the three-dimensional collimated self-accelerating beams should open a new degree of freedom for wave manipulations and have deep implications for various potential applications, especially in the fields of ultrasonic imaging, diagnosis and treatment. For instance, the beams may be used to generate negative radiation force to manipulate micro-particles. In additional, the metascreen may be employed to design novel ultrasonic transducers to overcome the “acoustic window” issue or deliver acoustic energy along designed arbitrary curvatures bypassing organs.

## Methods

Simulations are conducted with a commercial software based on finite elements method, COMSOL Multiphysics Version 5.1, in frequency domain with a fixed *λ* = 0.2 m. Considering the symmetry of the metascreen, two-dimensional axisymmetric models rather than three dimensional models are built for the simulations for reducing the calculating time. The HRs and the solid obstacles are made of steel with a density of 7800 kg/m^3^ and sound speed of 6100 m/s. The surrounding medium is air with its density 1.21 kg/m^3^ and sound speed 343 m/s. Perfectly matched layers are employed to mimic infinite space to obtain the sound fields shown in Figs 3 and 4. A plane wave with unit amplitude is employed as the incident wave in Fig. 3. A point source located at (*r*, *z*) = (0, −10*λ* − *h*) radiates a spherical wave in Fig. 4. The thermal dispassion and viscous loss are neglected in our simulations due to the fact the minimum width of the channels, *h*_2_, is ~61 times greater than the the thickness of the viscous boundary layers, 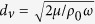, with *ω* and *μ* referring to angular frequency and the coefficient of dynamic viscosity. For higher frequencies, such as, 20000 Hz, *h*_2_ is just ~17 times bigger than *d*_*v*_ so that these effects need to be considered. The geometrical parameters of the elements should be re-optimized for good performance.

## Additional Information

**How to cite this article**: Li, Y. and Assouar, M. B. Three-dimensional collimated self-accelerating beam through acoustic metascreen. *Sci. Rep.* **5**, 17612; doi: 10.1038/srep17612 (2015).
